# Biogenically induced bedded chert formation in the alkaline palaeo-lake of the Green River Formation

**DOI:** 10.1038/s41598-019-52862-7

**Published:** 2019-11-11

**Authors:** Ryusei Kuma, Hitoshi Hasegawa, Koshi Yamamoto, Hidekazu Yoshida, Jessica H. Whiteside, Nagayoshi Katsuta, Masayuki Ikeda

**Affiliations:** 1 Graduate School of Environmental Studies, Nagoya University, Nagoya, 464-8601 Japan; 2 Faculty of Science and Technology, Kochi University, Kochi, 780-8520 Japan; 3 Material Research Section, University Museum, Nagoya University, Nagoya, 464-8601 Japan; 4 National Oceanography Centre Southampton, University of Southampton, Southampton, SO14 3ZH UK; 5 Faculty of Education, Gifu University, Gifu, 501-1193 Japan; 6 Graduate School of Science, Shizuoka University, Shizuoka, 790-8577 Japan

**Keywords:** Palaeoclimate, Geochemistry, Geology, Sedimentology

## Abstract

Rhythmically bedded cherts are observed in both pelagic marine and lacustrine deposits, but the formation mechanism in the latter remains highly uncertain. Our study of alternating chert–dolomite beds in the Eocene Green River Formation, Utah, USA reveals dense accumulations of organic-matter spheres (30–50 μm diameter) of probable algal cyst origin in the chert layers, and centennial- to millennial-scale periodicities in chert layer deposition. A positive correlation between the degree of degradation of the organic spheres and Si distribution implies decomposition of algal organic matter lead to precipitation of lacustrine chert. As both alkalinity and dissolved silica were likely high in the palaeo-lake waters of the Green River Formation, we hypothesize that decomposition of algal organic matter lowered the pH of sediment pore waters and caused silica precipitation. We propose a formation model in which the initial abundance of algal organic matter in sediment varies with productivity at the lake surface, and the decomposition of this algal matter controls the extent of silica precipitation in sediment. The formation of rhythmically bedded chert–dolomite may be linked to centennial- to millennial-scale climatic/environmental factors that modulate algal productivity, which are possibly tied to solar activity cycles known to have similar periodicities.

## Introduction

Rhythmically alternating beds of chert and shale, known as bedded chert, commonly occur in marine sedimentary rock of pelagic origin. Bedded chert in marine deposits consists mainly of biogenic silica, which originates from siliceous remains of radiolarians, sponges, and diatoms. Changes in the bed thickness of chert are interpreted to represent orbitally paced variations in marine productivity^1^ and/or the burial flux of biogenic silica in the deep-sea environment, the latter of which is controlled by the continental silicate weathering rate^2^. Bedded cherts exhibiting periodicity are also more rarely found in lacustrine deposits^3,4^; but due to the absence (or lack of preservation) of biogenic siliceous remains in pre-Neogene lacustrine chert, the formation mechanism of such bedded cherts remains far more uncertain.

Given the higher solubility of silica in alkaline waters with pH > 9, any process that causes variations in lake-water pH has the potential to be a major controlling factor in silica precipitation^5,6^. It is also noteworthy that lacustrine cherts occur bedded with either trona formed in an evaporative environment^3,4^ or with dolomite formed in a shallow saline-lake environment^7,8^. Upper Pleistocene lacustrine chert–trona deposits exposed around the highly alkaline Lake Magadi in the East African rift valley^3,9–12^ are considered the prime example of the first type, and similar deposits (referred to as Magadi-type cherts) have been reported from several other localities worldwide^4,13–15^. Previous studies concluded that dilution of alkaline brines by fresh-water input could decrease the pH of the lake water and result in precipitation of magadiite (NaSi_7_O_13_(OH)_3_·3H_2_O), which probably converted initially to kenyaite (NaSi_11_O_20.5_(OH)_4_·3H_2_O) and eventually to chert (6SiO_2_·H_2_O) by interaction with percolating waters. This inorganic precipitation process has been suggested for the lacustrine cherts in Lake Magadi^3^, but an alternative organically moderated precipitation mechanism has been suggested in a later study^16,17^ as described below.

Alternating beds of chert and dolomite have also been reported from lacustrine deposits^7,14,18^. Collinson^7^ reported thinly bedded chert, consisting of microcrystalline silica within micritic dolomite in middle Proterozoic strata of eastern North Greenland. The cherts were interpreted as having resulted from the precipitation of primary silica gels in response to the evaporative concentration of silica dissolved in groundwater. Wells^14^ reported nodular chert within the carbonate deposits of the Paleocene–Eocene Flagstaff Formation in northern Utah, USA, and also suggested that the cause of the initial silica precipitation was evaporative concentration. Buchheim^18^ reported the occurrence of chert nodules within dolomite beds in the Eocene Green River Formation, west-central USA, which formed in a shallow, hypersaline lake environment. Owen *et al*.^19^ observed spring-vent, pedogenic and shallow-marsh cherts close to fossil springs in the Kenya Rift. Many of these studies concluded that lacustrine chert is inorganic in origin, linked to the evaporative concentration of silica and/or subsequent precipitation by changes in the pH of the lake water^3,4^.

It remains intriguing, however, that fossils and biogenic textures are preserved in lacustrine chert^14^. Well-preserved biogenic materials (e.g., algae, pollen, spores, limnic organisms, and cyanobacterial remains) have been reported in lacustrine chert from the Green River Formation, USA^20^; the Paleogene succession of the Madrid Basin, Spain^8^; and Pleistocene deposits in Africa^17,21,22^. However, the potential role of the biogenic material and processes in the formation of lacustrine chert was not fully evaluated in previous studies.

A few studies have suggested a potential link between lacustrine chert formation and biogenic activity. Behr and Röhricht^16^ described evidence for calcareous bioherm and coccoid cyanobacteria structures in chert from Lake Magadi. They suggested that purely inorganic chert is rare in Lake Magadi, and instead proposed that the metabolic processes of cyanobacteria modify the pH of the pore water and influence silica precipitation. Hesse^4^ also documented several possible mechanisms of biogenic influence on silica precipitation, including production of CO_2_ by biogenic respiration in lake water or decomposition of organic matter that could result in lowering of the pH and dissolution of calcite and precipitation of silica. Although these studies describe potential linkages between lacustrine chert formation and biogenic activity, the mechanism by which biogenic activity results in the precipitation of silica still remains unclear. Specifically, clear evidence of the biological activity that produced CO_2_ and pH changes to drive silica precipitation have yet to be demonstrated.

Here we present field observations and geochemical evidence from the Eocene Green River Formation in northern Utah, USA, that indicate deposition of organic matter and its decomposition might play a key role in the formation of lacustrine chert. We find dense accumulations of spherical organic materials of probable algal origin in chert beds, and infer that decomposition of this organic material drove silica precipitation by decreasing the pH of pore waters in bottom sediments. We also discuss the possibility that changes in algal productivity of the lake gave rise to the periodicity observed in lacustrine bedded chert.

## Bedded Chert in the Green River Formation

**Figure 1:**
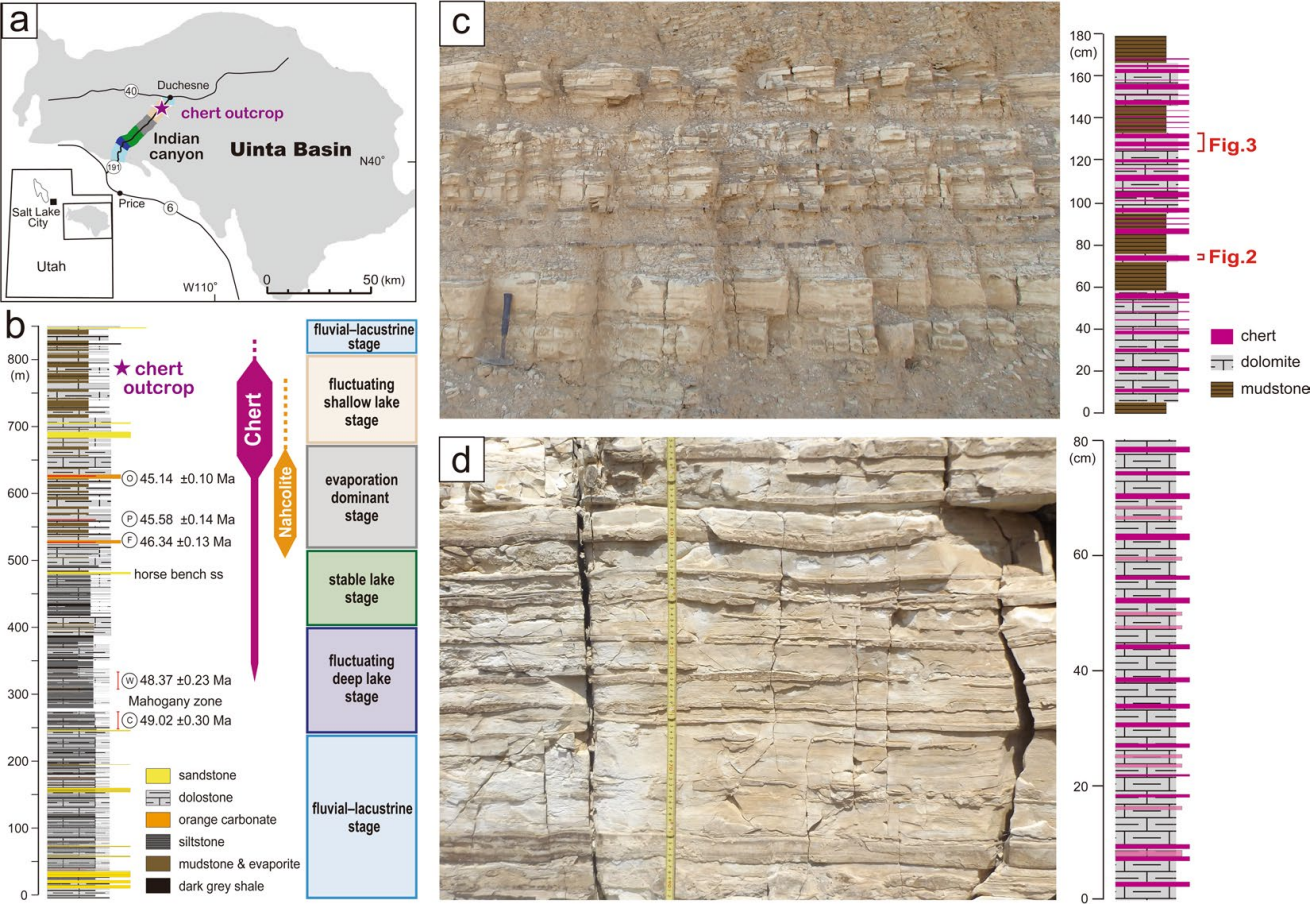
Location map, division of lacustrine paleonenvironments, lithostratigraphic columns, and outcrop photographs of the bedded chert succession in the Green River Formation. (**a**) Location map of the Green River Formation in the Indian Canyon section, western Uinta Basin. (**b**) Lithostratigraphic column, chert occurrences, and subdivision of lacustrine palaeoenvironments; ^40^Ar/^39^Ar ages of intercalated tuffs are from Smith *et al*.^26^ O: Oily tuff; P: Portly tuff; F: Fat tuff; W: Wavy tuff; C: Curly tuff. (**c**,**d**) Outcrop photographs and lithological columns of alternating beds of chert, dolomite, and mudstone. Occurrences of chert beds represent marked periodicities of centimetre-scale changes in thickness. All photographs (**c,d**) shown here are taken by R. Kuma.

Lower to middle Eocene lacustrine deposits of the Green River Formation are widely distributed in the central part of the United States, such as the Greater Green River Basin in Wyoming, the Uinta Basin in northern Utah, and the Piceance Creek Basin in Colorado^23–25^. We examined the lacustrine bedded chert succession in the upper part of the formation in the Indian Canyon section, western Uinta Basin (Fig. 1a). The thickness of the Green River Formation varies in each basin^25^, and is about 850 m thick in the Indian Canyon section. Deposition of the lacustrine sediments occurred over a period of 9 Myr, between ca. 53 and ca. 44 Ma^26,27^. The estimated sedimentation rate of the formation in the Indian Canyon section is ca. 9–10 cm/kyr, based on ^40^Ar/^39^Ar ages of intercalated tuffs^24^.

Following the stratigraphic framework of previous studies^20,26,28,29^, we subdivided the succession of the Indian Canyon section into six stages on the basis of lithology. In ascending stratigraphic order, these are the fluvio-lacustrine, fluctuating deep-lake, stable lake, evaporation-dominant, and fluctuating shallow-lake stages (Fig. 1b). Cherts alternate with dolomite beds and occur mainly in the fluctuating shallow-lake stage (Fig. 1c,d). The occurrences of chert layers show marked periodicities corresponding to thicknesses of ca. 7–9 and 17–20 cm (Supplementary Fig. S1). Microscopic observations reveal that the dolomite beds have a micritic texture, with no clear sign of replacement texture. Due to similarities with modern lacustrine dolomite^30^, dolomite beds in the Green River Formation are interpreted to have been formed by saturation-induced primary precipitation in a shallow saline-lake environment^31^. Previous studies also suggested that chert-bearing beds of the Green River Formation are enriched in Si, Mg, and Na, which indicate deposition in highly alkaline (pH > 9) paleoenvironment^18,32,33^. Based on this evidence, the chert–dolomite deposits are interpreted to have been deposited in a highly alkaline and saline-lake palaeoenvironment. Given the occurrence of evaporitic minerals (i.e., trona, nahcolite, and dolomite), cherts in the Green River formation have been identified as Magadi-type chert and attributed to an inorganic evaporative origin^4,34,35^. However, in light of the contrary ideas put forward by Behr and Röhricht^16^, we sought observational and geochemical evidence that would help evaluate if chert formation was related to the deposition and decomposition of organic matter.

## Abundant Organic Spheres in Chert Layers

**Figure 2:**
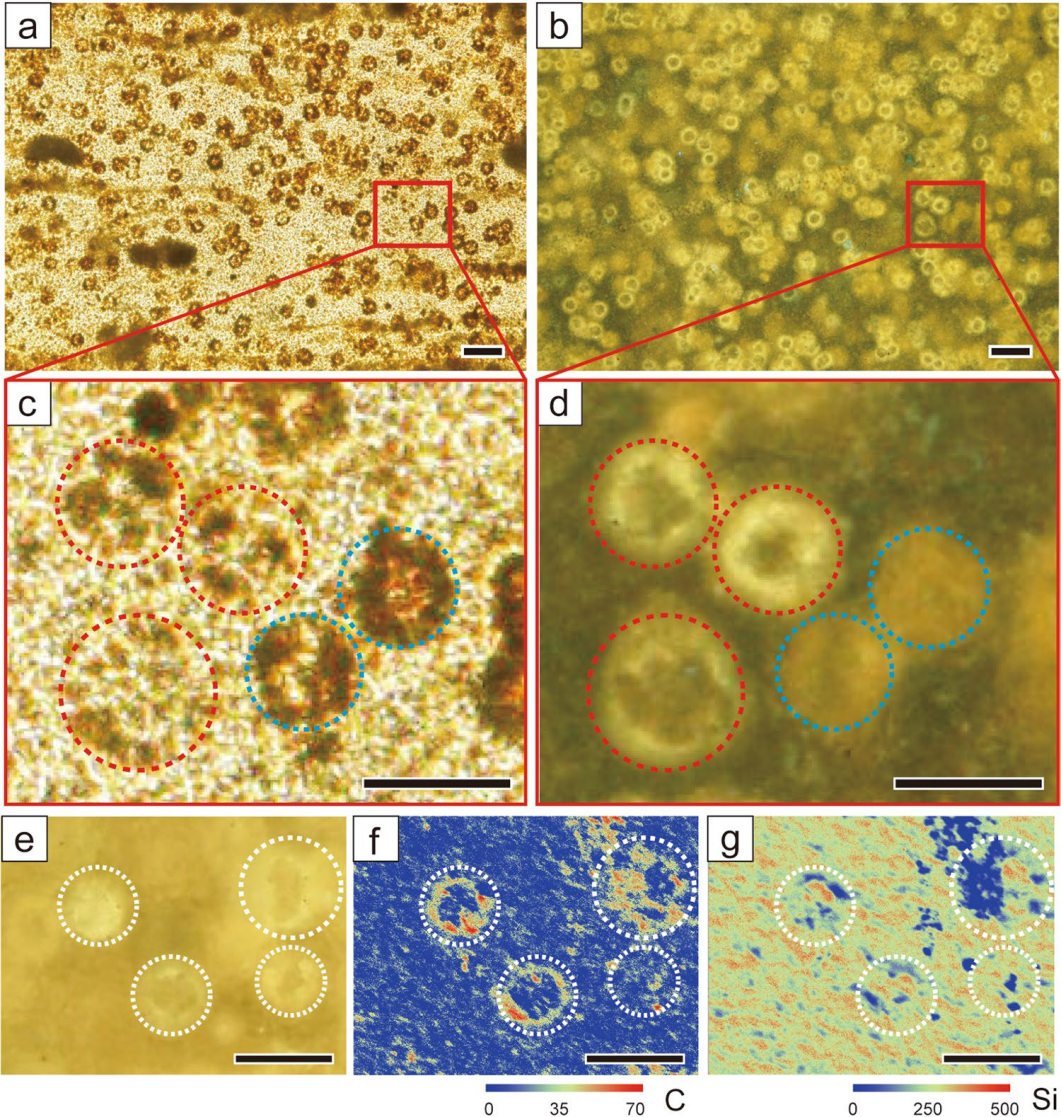
Photomicrographs of, and elemental distributions of, a chert bed from (**a**) optical microscopy and (**b**) fluorescent microscopy. (**c**,**d**) Enlarged photograph of the area indicated by a red square in (**a**,**b**). Some organic spheres are visible in optical microscopy (**c**), but not clear under the fluorescent microscope (**d**) (indicated by a blue dotted circle). In contrast, the strongly fluorescent organic spheres (**d**) are not as visible under the optical microscope (**c**) (indicated by red dotted circle). (**e**) Detailed fluorescence photograph and XRF maps of (**f**) C and (**g**) Si. High-C concentration in the outer part of organic spheres while high-Si concentrations occur both inside and outside of organic spheres. Scale bars in (**a,b**) and (**c–g**) are 100 μm and 50 μm, respectively.

Under the optical and fluorescent microscopes, we found dense accumulations of spherical materials with a limited size range (30–50 μm) in the chert layers (Fig. 2). These include less- and non-fluorescent spheres only visible in optical microscope, and highly fluorescent spheres that are barely visible under the optical microscope (Fig. 2c,d). Elemental mapping by scanning electron microscopy and energy dispersive X-ray spectroscopy (SEM-EDS) reveals that the highly fluorescent spheres are composed of organic-carbon-encrusted shells, and Si is distributed both inside and outside the shells (Fig. 2e–g). Given that algal organic matter contains polycyclic aromatic hydrocarbons and exhibits strong fluorescence^36^; these spheres have the characteristics of organic matter of algal origin, and a morphology resembling the green algae *Botryococcus braunii*^37,38^ or chrysophytes^39^. Biomarker analyses further suggest a higher contribution of lacustrine algae and bacteria in the chert layers (Supplementary Fig. S5). Since the size and morphology of both the non-fluorescent and highly fluorescent organic spheres are similar, we interpret the spheres as originally representing a single species, but that the variable degradation of organic matter led to their differences in optical visibility.

**Figure 3:**
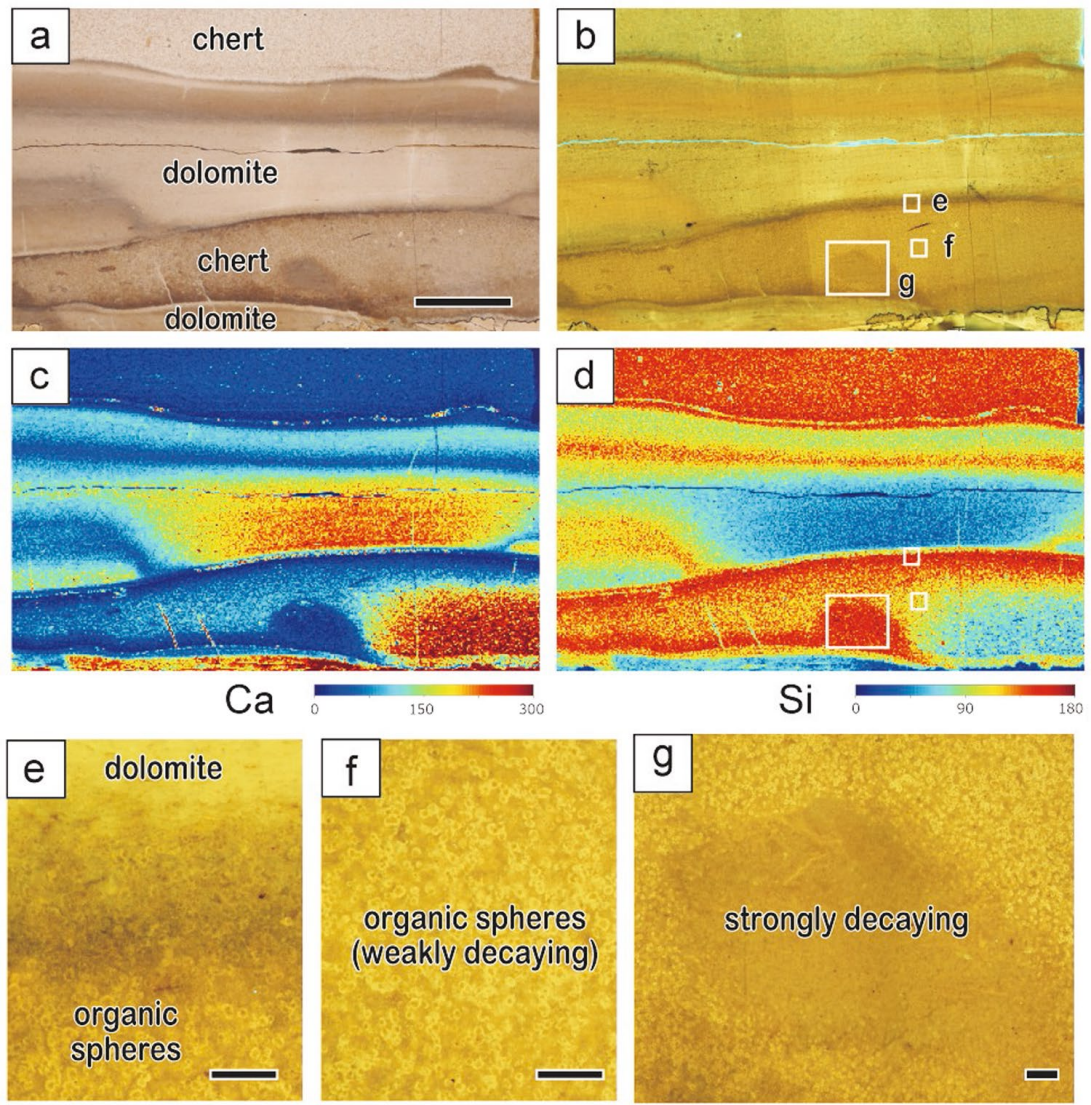
Successive photomicrographs and XRF images of a bedded chert sample. (**a**) Optical and (**b**) fluorescent photomicrograph. (**c,d**) XRF images. (**e–g**) Detailed fluorescence photographs of the areas indicated by white squares in (**b**). Scale bars in (**a–d**) and (**e–g**) are 1 cm and 500 μm, respectively.

To clarify the relationship between organic sphere occurrence, degradation degrees, and Si concentration, we undertook elemental mapping by scanning X-ray analytical microscope (SXAM) (Fig. 3). The high-Si chert beds and high-Ca dolomite beds are separated by sharp boundaries. The strongly fluorescent organic spheres are clearly observed in chert beds, but not in dolomite beds. In addition, the degradation degree of algal organic matter seems to correlate with the heterogeneous Si concentration within a chert layer. Strongly fluorescent and weakly degraded organic spheres are abundant in regions with slightly lower Si concentrations, whereas such organic spheres are less apparent in areas with higher Si concentrations, such as near the boundaries with dolomite beds (Fig. 3). Based on the modal composition analysis of organic spheres, the percentage of “visible” organic spheres are more than 90% in weakly degraded areas, but only about 10% in strongly degraded areas (Supplementary Figs S2; S4).

In addition to periodicities corresponding to thicknesses of ca. 7–9 and 17–20 cm in the chert layers (Supplementary Fig. S1), elemental mapping by SXAM confirms that chert and dolomite beds alternate on a scale of ca. 1.0–1.2 and 2.2–3.0 cm (Supplementary Fig. S3). X-ray fluorescence (XRF) analyses reveal that SiO_2_ comprises about 90% of the chert samples, CaO about 3%, and Al_2_O_3_ < 0.01%, a finding that indicates alumino-silicates are not a major contributor to Si in the chert.

## Discussion

Previous studies have linked the formation of lacustrine chert to the inherently high silica solubility in alkaline waters and the precipitation of silica driven by changes in the pH in lake waters^3^ and/or sediment pore waters^4^. In the case of the Green River Formation, the presence of nahcolite and trona indicate the lake water had a high pH of >9^18,31,32^, similar to Lake Magadi^11^. The lake water presumably acquired its alkalinity and dissolved silica from weathering of surrounding intercalated volcaniclastic rocks that are widely distributed in the Colorado Plateau^26,40,41^. Therefore, evidence for highly alkaline lake waters and for adequate sources of silica during deposition of the Green River Formation is clear, but the mechanism that lowered the pH of lake or sediment pore waters and led silica precipitation is uncertain.

We present evidence that the distribution of high-Si concentration corresponds to dense accumulations of spherical organic matter (Figs 2 and 3). The size and morphology of these spheres resemble those of the green algae *Botryococcus braunii*^38^, which are widely reported in the Green River Formation^42–44^, or chrysophytes as commonly observed in oil shale^39^. As no siliceous organisms (e.g., diatoms) have been found in the Green River Formation, Si precipitation by such organisms is unlikely. The size and morphology of observed organic spheres are also quite different from lacustrine diatoms of Middle Eocene age reported in Canada^45^ and Upper Cretaceous age in Mexico^46^. Instead, the presence of abundant organic spheres in chert layers implies that deposition of algal organic matter was integral to formation of lacustrine chert. Biomarker evidence also supports the formation of chert layers by algal organic matter influence (Supplementary Fig. S5). *Botryococcus* is generally prevalent under oligotrophic lacustrine conditions^38,47^, consistent with the observed predominance of bedded cherts in the shallow-lake environmental facies of the Green River Formation.

**Figure 4:**
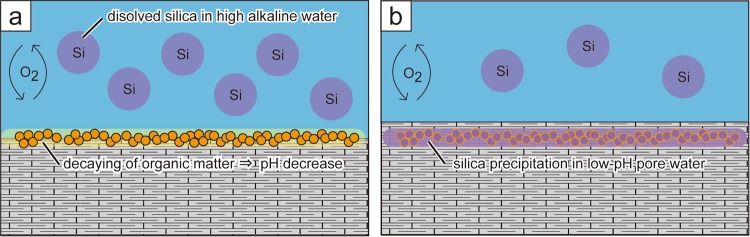
Schematic illustrations of the mechanism of lacustrine chert formation in the Green River Formation. (**a**) Abundant algal organic spheres were initially deposited within dolomite-rich sediments in the shallow and highly alkaline lake rich in dissolved Si. Subsequently, the algal organic spheres were decomposed and the pH of pore water in the sediment around the organic spheres became acidic. (**b**) The decreased pH of the pore waters would then cause the abundant dissolved silica to precipitate around the decomposed organic spheres.

Based on these lines of evidence, we propose the following mechanism for the formation of lacustrine bedded chert in the Green River Formation (Fig. 4). Abundant algal organic spheres were initially deposited within dolomite-rich sediments on the shallow saline-lake bottom; subsequently, algal organic spheres underwent variable decomposition and the surrounding pore waters became acidic. The decreased pH of the pore waters would then cause the abundant dissolved silica to precipitate around the decomposed organic spheres. The observed relationship between heterogeneous Si concentration and fluorescence/visibility of organic spheres also supports the idea that degradation of algal organic matter in bottom sediment is what drove silica precipitation in an early diagenetic stage. The observed dehydration structures (Supplementary Fig. S6), which imply brittle to brittle-ductile deformation origin, also suggest Si precipitation and lithification would occur in early diagenetic stage.

This proposed formation model is consistent with the relationship between lithofacies and occurrences of bedded chert. Bedded cherts occur mainly in the fluctuating shallow-lake stage, and are less abundant in the stable lake stage and evaporation-dominant stage and almost absent in the fluvio-lacustrine and fluctuating deep-lake stages (Fig. 1). The alkalinity and dissolved silica content of the lake water are assumed to have been lower in the fluvio-lacustrine and fluctuating deep-lake stages, and the lower dissolved Si content in those lake waters might prevent chert formation. In addition, the reducing environment of the lake bottom (abundant pyrite grains of phytoclast origin are observed in the deep-lake facies, and are likely to have been formed by sulphate reduction of higher plant materials) would have suppressed the decomposition of organic matter, so any lowering of pH by this mechanism would be unlikely during the deep-lake stage. Conversely, decomposition of organic matter is likely to occur under the oxidising conditions of a shallow lake (Fig. 4). Therefore, we infer that silica precipitation, facilitated by the decomposition of algal organic matter, was predominant only in the shallow-lake environmental facies. In the case of the evaporation-dominant stage, the lake level was too shallow/ephemeral to support an algal habitat.

In contrast to our model, most previous studies have attributed the formation of lacustrine chert to inorganic processes and evaporative precipitation in a shallow-lake environment^3,4^. Evaporative concentration remains a possible mechanism for silica precipitation, but the documentation of cyanobacterial metabolic processes in the “type” lacustrine bedded chert deposits at Lake Magadi^16^ and the growing evidence for biogenic structures within many other bedded chert deposits of modern to ancient age^20,21,48^ demands a careful evaluation of the role of biologic activity. A key issue is that for biogenically induced chert formation, the relationship between chertification mechanisms and preserved biogenic signatures is not well documented (i.e., did pH lowering result from biogenic respiration or decomposition of organic matter?). For the Green River formation, we have shown that significant organic matter is present and that the degree of organic matter decomposition in bottom sediment is spatially and temporally closely tied to the formation of lacustrine bedded chert. Future studies will have to evaluate if this formation mechanism can be successfully applied to other chert deposits that preserve biogenic structures^16,20,21,48^.

A similar model of biogenically induced silica precipitation has been suggested in studies of pedogenic rhizoliths and ichnofossils^22,49^. Owen *et al*.^49^ reported siliceous rhizoliths in Pleistocene deposits in Kenya that were possibly formed by plant-root decomposition and the resulting lowering of pH, with the deposited silica sourced from plant opal. Buatois *et al*.^22^ described the siliceous ichnofossil *Vagorichnus* in the sub-lacustrine hydrothermal deposits of Lake Baringo, central Kenya, where they interpreted silica precipitation to have occurred as a result of the interplay between the decomposition of organic matter and dissolved silica sourced from hydrothermal deposits.

The causal mechanism underlying the periodic alternations of chert and dolomite also needs further study. If lacustrine bedded chert is formed by biogenically induced decomposition of algal organic matter, as is proposed in this study, what process accounts for the apparent periodicity of chert deposition? From the field investigations and elemental mapping analysis, alternating beds of chert and dolomite exhibit periodicities in thickness of ca. 1.0–1.2, 2.2–3.0, 7–9, and 17–20 cm (Supplementary Figs S1, S3). With an estimated sedimentation rate of ca. 9–10 cm/kyr for the Indian Canyon section, the chert occurrences correspond to estimated periodicities of about 100–130, 220–330, 700–1000, and 1700–2200 years. It is therefore possible that centennial- to millennial-scale changes in lake algal productivity modulated the availability of algal organic matter in lake-bottom sediments, and ultimately the abundance of chert that could be precipitated. It is also noteworthy that these calculated periodicities resemble the hierarchy of well-documented solar activity cycles (e.g., the 88–105-year Gleissberg cycle, the 210–230-year de Vries cycle, the 1000-year Eddy cycle, and the 2000–2300-year Hallstatt cycle)^50^. Therefore, solar activity cycles, which are known to influence climatic change^51^, appear to be implicated as a control on the centennial- to millennial-scale changes in algal productivity, although further investigation is required to test this hypothesis.

## Methods

To perform microscopic observations and geochemical analysis, we collected samples of alternating chert–dolomite. Occurrence periodicities of bedded chert involved measuring the thickness variations of alternating beds of chert and dolomite from a well-exposed succession (Supplementary Fig. S1) at Indian Canyon, Utah (GPS coordinates 40°7′32.60″N, 110°26′31.80″W). Optical and fluorescent photomicrographs of chert samples were taken in the Faculty of Science and Technology of Kochi University (OLYMPUS BX51). SEM-EDS analysis (HITACHI SU6600 and EMAX x-act) for elemental mapping and XRF analysis (Rigaku ZSX Primus II) for major elemental compositions were carried out in the Graduate School of Environmental Studies of Nagoya University.

SXAM analysis was conducted to show semi-quantitatively the two-dimensional distribution of elements Si, and Ca across the entire surface of samples, using an XRF microscopy (Horiba, XGT-5000) in the Nagoya University Museum (Supplementary Figs S6; S7). A high-intensity continuous x-ray beam (Rh anode, 50 kV/1 mA), 100 mm in diameter, was focused with a guide tube and irradiated perpendicular to the surface of the samples, which were placed on a PC-controllable X-Y stage. Two-dimensional distributions of Si content obtained from counting data of SXAM were converted into one-dimensional element profile in a direction perpendicular to the alternating chert–dolomite beds (Supplementary Figs S6; S7)^52^. Time series analysis for one-dimensional Si content was performed using AnalySeries software^53^. The details of time series analysis are described in supplementary material (Supplementary Figs S1, S3).

Source input and paleodepositional conditions were interpreted based on the molecular composition of four chert samples^54,55^. Samples were powdered and subsequently subjected to Accelerated Solvent Extraction of total lipid extracts followed by evaporation, fractionation using liquid chromatography into aliphatic (saturated) hydrocarbon, aromatic hydrocarbon and polar compound aliquots. The saturated fractions were further analysed using a Thermo Trace 1310 gas chromatograph coupled to a Thermo TSQ8000 mass spectrometer at University of Southampton.

## Supplementary information

Supplementary Information

## Data Availability

All the data reported in this article are available from the corresponding author.
