# Supplementary material for: Biogenically induced bedded chert formation in the alkaline palaeo-lake of the Green River Formation
Source: Sci Rep. 2019 Nov 11;9:16448. doi: 10.1038/s41598-019-52862-7 (PMC6848130; doi:10.1038/s41598-019-52862-7)
Supplement: Supplementary file 1 — Supplementary Information [file 41598_2019_52862_MOESM1_ESM.pdf]

Biogenically induced bedded chert formation in the alkaline palaeo-lake of the Green River Formation

Supplementary Data

Ryusei Kuma[1]*, Hitoshi Hasegawa[2], Koshi Yamamoto[1], Hidekazu Yoshida[3], Jessica H. Whiteside[4], Nagayoshi Katsuta[5], Masayuki Ikeda[6]

[1] Graduate School of Environmental Studies, Nagoya University, Chikusa, Nagoya, Japan.
*corresponding author kuma.ryusei@i.mbox.nagoya-u.ac.jp
[2] Faculty of Science and Technology, Kochi University, Akebono, Kochi, Japan.
[3] Material Research Section, University Museum, Nagoya University, Chikusa, Nagoya, Japan.
[4] University of Southampton, National Oceanography Centre Southampton, Southampton, UK.
[5] Faculty of Education, Gifu University, Yanagido, Gifu, Japan.
[6] Graduate School of Science, Shizuoka University, Suruga, Shizuoka, Japan.

The supplementary data provided here cover the details of outcrop occurrence of bedded chert, time series analysis periodicities of chert beds, the results of modal composition analysis and SXAM analysis, and molecular geochemistry.

Time series analysis for chert occurrences in outcrop (Fig. S1) and Si content (Fig. S2)

To examine the sedimentary rhythms of the bedded chert, the thickness of individual chert beds, dolomite, and mudstone beds were measured at the outcrop (Supplementary Fig. S1). We interperted the thickness of chert beds to represent the initial abundance of organic matter in sediment, and the thickness of dolomite bed to represent intervals with low algal productivity, assuming dissolved silica contents in lake water and sedimentation rate were constant. To detect the relative amplitudes of thickness variations in chert layers, we performed band-pass filtering using a Gauss algorithm in the software AnalySeries to extract dominant spectral peaks[51]. The interpolated data were subjected to Fourier analysis using a FFT option within AnalySeries and Bartlett window with the linear trend removed to obtain the power spectra (Periodogram and B-Tukey). The band-widths of the filters were selected to capture the spectral power associated with the dominant cyclicities. The thickness variations of alternating beds of chert and dolomite show marked periodicities of ca. 7–9, and 17–20 cm (frequency = 0.125; bandwidth = 0.04 and frequency = 0.054; bandwidth = 0.008).

Elemental mapping analysis by SXAM was performed for an interval of chert and dolomite beds. Two-dimensional distributions of Si content obtained from counting data of

SXAM were converted into a one-dimensional element profile in a direction perpendicular to the alternating chert–dolomite beds (Supplementary Fig. S3)[50]. Selected areas of Si profiles are also shown in Supplementary Fig. S3 and S4. The obtained one-dimensional Si profile revealed that chert and dolomite bed alternations show periodicities on a scale of ca. 1.0–1.3 and 2.2–3.0 cm (frequency = 0.09; bandwidth = 0.03 and frequency = 0.038; bandwidth = 0.015).

On the basis of these field observations and the elemental mapping analysis, alternating beds of chert and dolomite exhibit periodicities in thickness of ca. 1.0–1.2, 2.2–3.0, 7–9, and 17–20 cm (Supplementary Figs. S1, S3). Given that the estimated sedimentation rate of the formation in the Indian Canyon section is ca. 9–10 cm/kyr, the chert occurrences correspond to periodicities of about 110–120, 260–290, 700–1000, and 1700–2200 years. Thus, the periodic occurrences of chert beds in the Green River Formation are possibly linked to centennial- to millennial-scale changes in lake algal productivity that are possibly modulated by solar activity cycles[48].

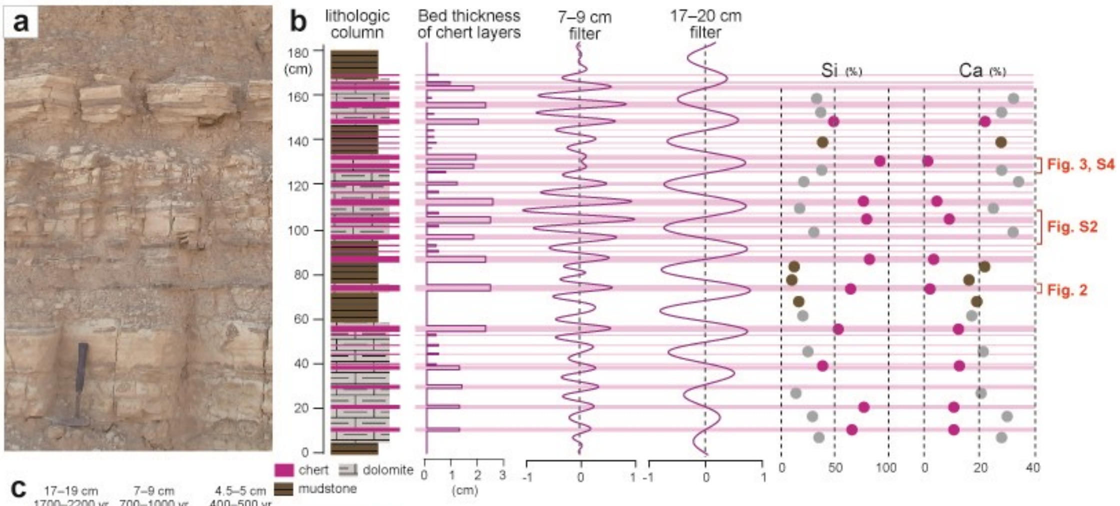

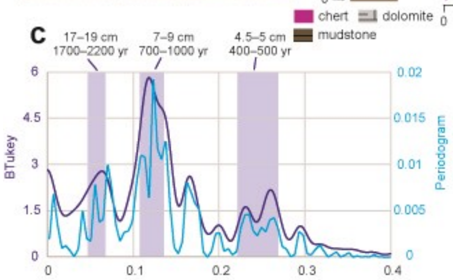

Supplementary Figure S1. Time series analysis of thickness of chert layers. (**a**) outcrop photographs, (**b**) lithologic column of alternating beds of chert, dolomite and mudstone, thickness variations of chert layers, and its amplitude modulation of 7–9 and 17–20 cm periodicities. Also shown are Si and Ca contents measured by Handy XRF analyser (Oxford, X-MET 8000). (**c**) FFT analysis (Periodogram and B-Tukey methods) of bed thickness variations of chert layers. The photograph-shown here is taken by R. Kuma

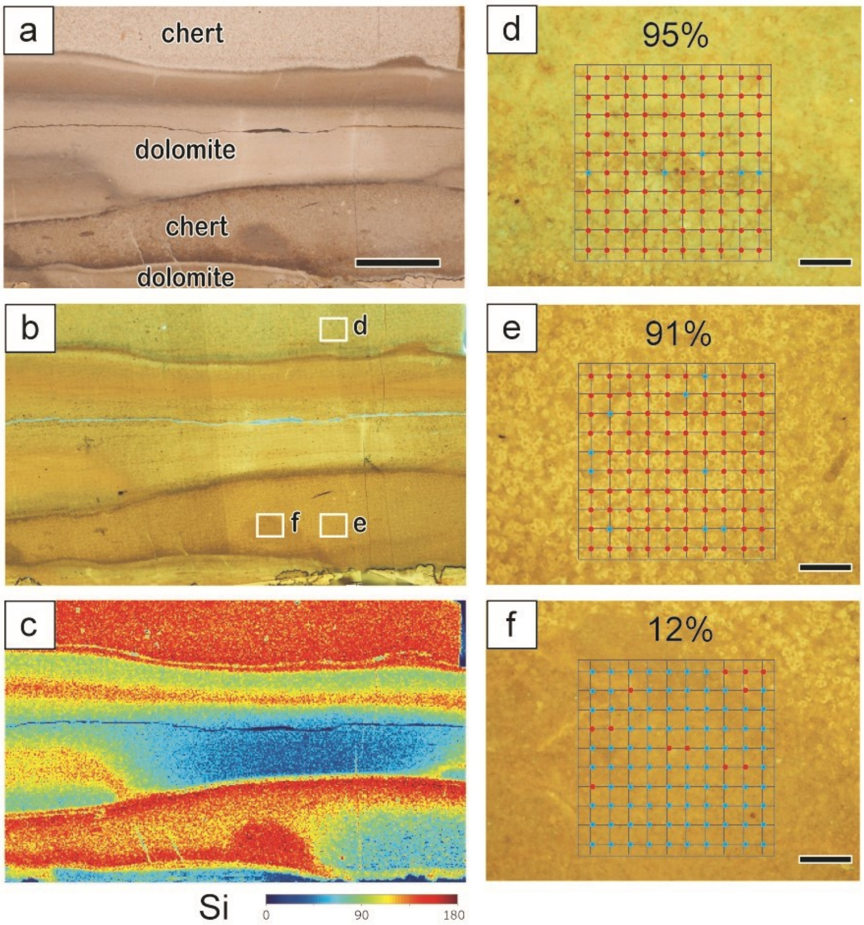

Supplementary Figure S2. Modal composition analysis of organic spheres. (**a**) Optical and (**b**) fluorescent photomicrograph. (**c**) XRF image. (**d–f**) Detailed fluorescence photographs and the percentage of “visible” organic spheres of the areas indicated by white squares in (**b**). Red dots indicate the point of visible organic spheres and blue dots indicate absence of organic spheres. Scale bars in (**a–c**) 1 cm and (**d–f**) 500 μm, respectively.

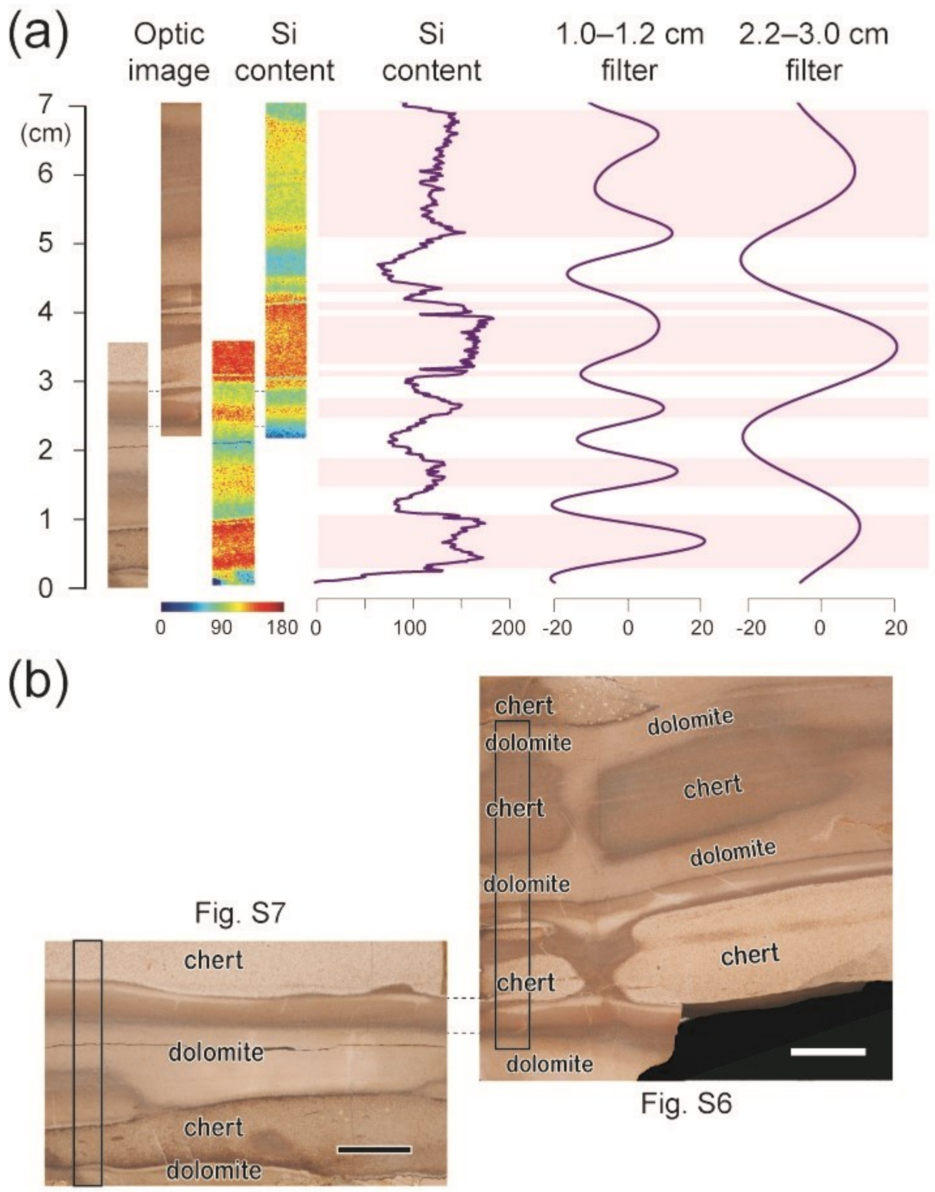

Supplementary Figure S3. (**a**) The Si profile variations of alternating beds of chert and dolomite show marked periodicities of ca. 1.0–1.2 and 2.2–3.0 cm in thickness. (**b**) The relationship between the selected Si profile of alternating beds of chert and dolomite is indicated by the black rectangles in Figs. S6 and S7.

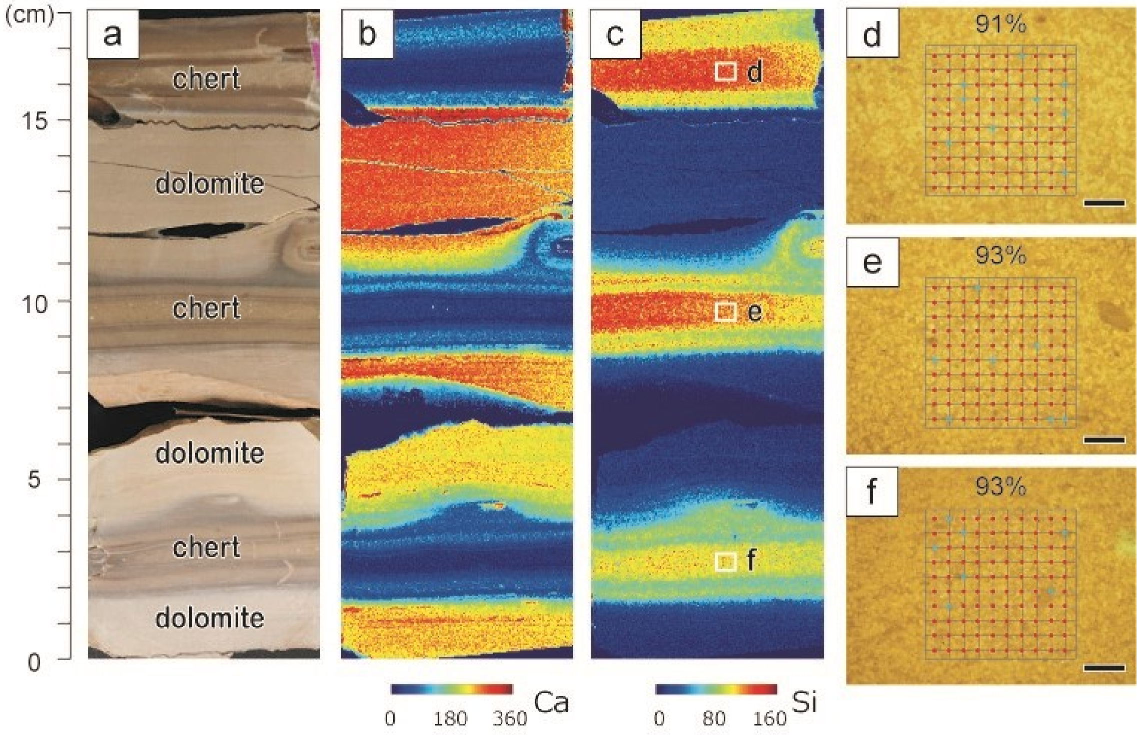

Supplementary Figure S4. (**a**) Optical and (**b–c**) XRF images. (**d–f**) Detailed fluorescence photographs and the percentage of "visible" organic spheres of the areas are indicated by white squares in (**b**). Red dots demarcate the point of visible organic spheres and the blue dots mark the absence of organic spheres. Scale bars in (**d–f**) 500 µm, respectively.

Molecular Geochemistry of the organic matter

Four samples from chert beds were analysed to evaluate their source input and paleodepositional conditions. The distributions of diasteranes and steranes (C27–C29) are shown in Supplementary Fig. S5a. The sterane composition can be used to provide an indication of source differences[54]. C27 sterols (steranes) mainly derive from algae, while the C29 sterols are more typically associated with land plants (although microalgae or cyanobacteria can also be important sources of C29 sterols)[55]. Low C28 levels are typical of limnic environments[55], and support *Botryococcus* or chrysophytes as the main phytoplankton source[37] for the chert samples, with additional influence of cyanobacterial remains. Low to moderate hopane/sterane ratios (2–3) and the presence of gammacerane support this source input interpretation, and in concert with low Pr/Phy ratios, indicate hypersaline depositional conditions (Supplementary Fig. S5b) that supported a highly productive community.

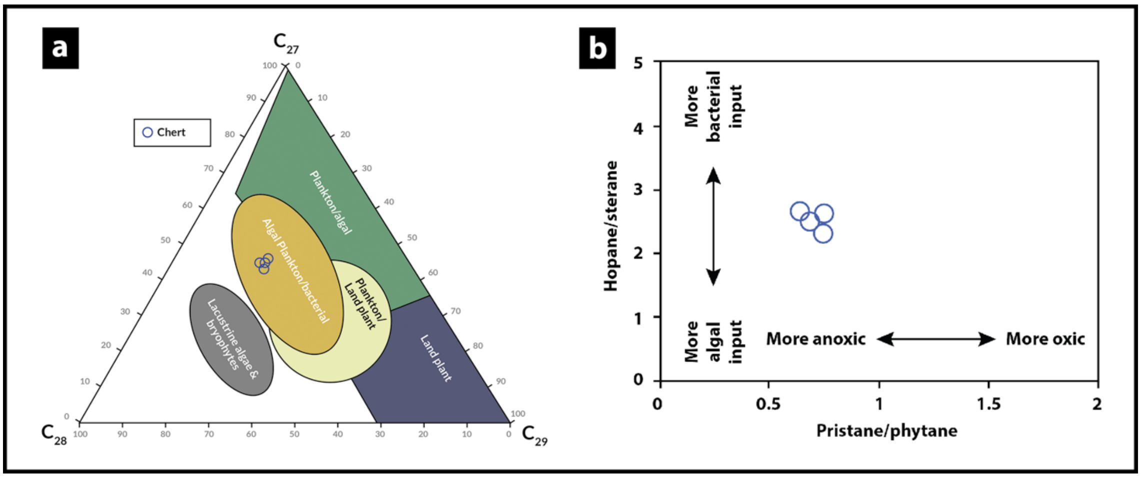

Supplementary Figure S5. Source input and paleodepositional conditions interpreted based on the molecular composition of four chert samples from the Green River Fm. (a) Ternary diagram of regular steranes (C27, C28 and C29) showing the relationship between sterane compositions and organic matter input (**b)** Cross-plot of hopane/sterane ratios versus pristane/ phytane ratios suggesting deposition in a hypersaline environment.

SXAM analysis results (Figs. S6, S7)

The SXAM intensity maps of alternation of chert and dolomite were measured to show semiquantitatively the two-dimensional distribution of elements Si, Ca, P, S, and Fe across the whole surface of the samples (upper sample: Fig. S6, lower sample: Fig. S7).

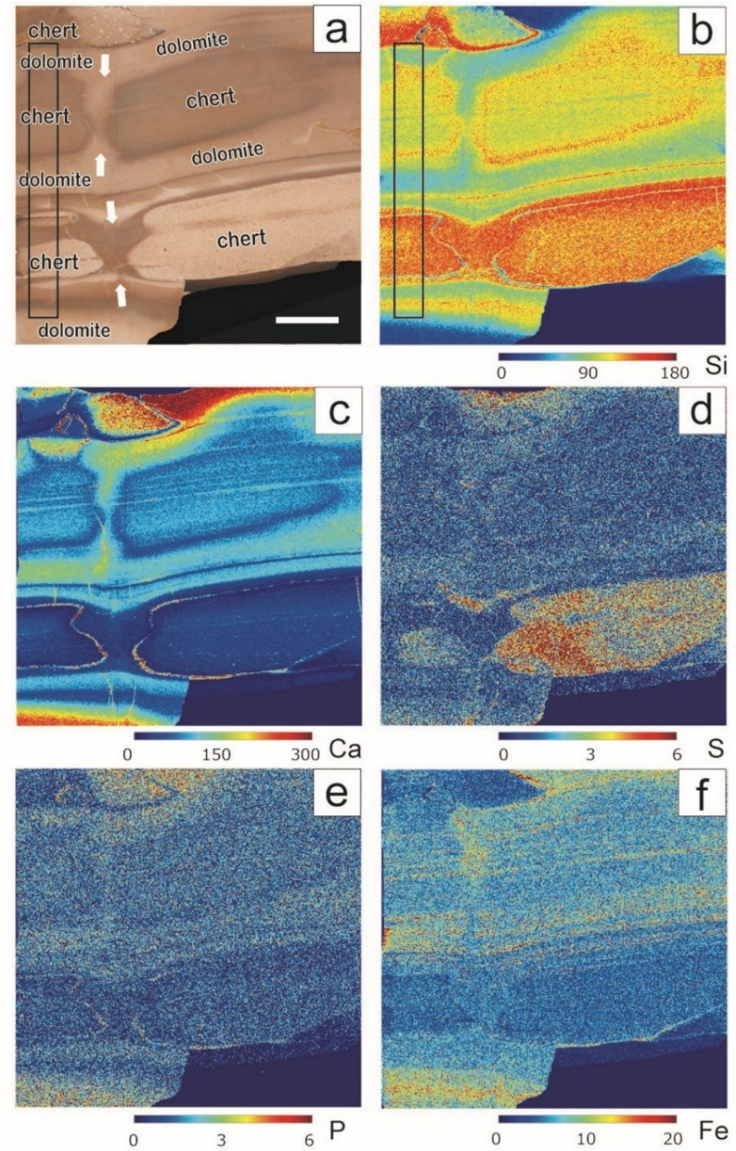

Supplementary Figure S6. Successive photomicrograph and XRF images of a bedded chert sample (upper). (**a**) Optical photomicrograph with dehydration structures (white arrows). (**b–f**) XRF images. Scale bar is 1 cm.

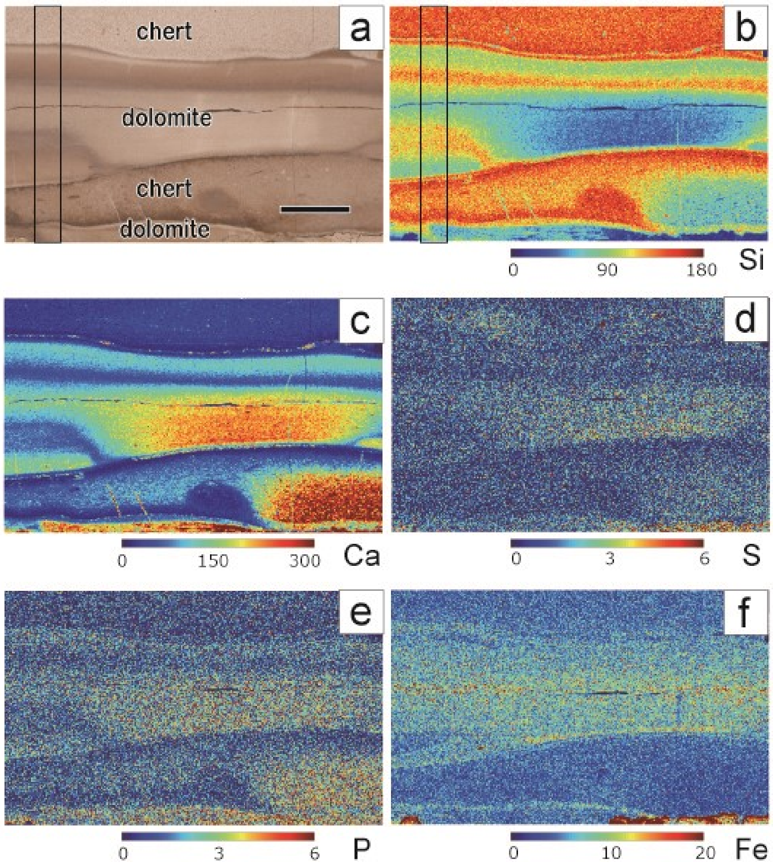

Supplementary Figure S7. Successive photomicrograph and XRF images of a bedded chert sample (lower). (**a**) Optical photomicrograph. (**b–f**) XRF images. Scale bar is 1 cm.
